# Real-world effectiveness of eptinezumab in chronic migraine—increase in good days in three subgroups: psychiatric comorbidities, prior subcutaneous anti-calcitonin gene-related peptide therapy, and migraine-associated brain fog

**DOI:** 10.1186/s10194-025-02165-2

**Published:** 2025-11-27

**Authors:** Charles Argoff, Fawad A. Khan, Steven P. Herzog, Ryan M. Smith, Seema Soni-Brahmbhatt, Divya Asher, Susanne F. Awad, S. Wald Grossman, Foram Patel, Dawn C. Buse

**Affiliations:** 1 Albany Medical Center, 47 New Scotland Ave, Albany, NY 12208 USA; 2 The McCasland Family Comprehensive Headache Center, Ochsner Neurosciences Institute, Ochsner Health, New Orleans, LA USA; 3 The University of Queensland Medical School, Brisbane, Australia; 4 Texas Neurology, Dallas, TX USA; 5 St. Luke’s Health System, Meridian, ID USA; 6 Lundbeck LLC, Deerfield, IL USA; 7 H. Lundbeck A/S, Copenhagen, Denmark; 8 Department of Neurology, Albert Einstein College of Medicine, Bronx, NY USA

**Keywords:** Chronic migraine, Real-world, Eptinezumab, Prior preventive treatment failures, Brain fog, Cognition, Depression, Anxiety, Anti-calcitonin gene-related peptide, Monoclonal antibody

## Abstract

**Background:**

The REVIEW study evaluated the real-world experiences of individuals treated with eptinezumab for chronic migraine (CM) in the outpatient setting. This post hoc analysis explored eptinezumab effectiveness in three participant subgroups: those who had self-reported psychiatric comorbidities, those previously treated with subcutaneous anti-calcitonin gene-related peptide (anti-CGRP) monoclonal antibodies (mAbs) for migraine prevention, or those who had reported ever experiencing symptoms of brain fog.

**Methods:**

Adults with a CM diagnosis who completed ≥ 2 eptinezumab infusion cycles provided survey responses that included the number of good days (participant-defined) per month before and after initiating eptinezumab, the presence/absence of comorbid psychiatric conditions, the number and type of subcutaneous anti-CGRP mAbs used prior to initiating eptinezumab, and the presence/absence of brain fog (feeling confused, difficulty learning/remembering, or trouble speaking/reading) and its improvement after initiating eptinezumab.

**Results:**

Among 94 participants, 61 (65%) reported psychiatric comorbidities, and 84 (89%) had previously used a subcutaneous anti-CGRP mAb. Average increase from baseline in participant-reported total number of good days per month after initiating eptinezumab were 9.8 and 10.1 days for those with the presence or absence of psychiatric conditions at baseline, respectively; based on the type of subcutaneous anti-CGRP mAb, average increase from baseline were, 9.2 days (erenumab), 10.6 days (fremanezumab), and 10.0 days (galcanezumab); and based on the number of prior mAbs, 9.9 days (0 prior), 8.9 days (1 prior), 11.7 days (2 prior), and 8.6 days (3 prior). Participants reporting complete/very much improvement in brain fog had an average 15-day increase in the number of good days per month, while in participants that reported brain fog did not improve, there was a 1-day increase.

**Conclusions:**

This post hoc analysis of REVIEW showed that eptinezumab treatment can provide patient-perceived benefits across a spectrum of individuals with CM, including those with psychiatric comorbidities, in real-world settings. A greater number of good days regardless of number of prior mAb therapy supports the use of eptinezumab earlier for individuals with CM, rather than cycling through multiple anti-CGRP therapies. Furthermore, good days can provide a meaningful and measurable endpoint to assess preventive treatment and is correlated with improvements in brain fog.

**Supplementary Information:**

The online version contains supplementary material available at 10.1186/s10194-025-02165-2.

## Background

Based on epidemiological population-based studies, it is estimated that around 16% of the adult population in the United States (US) has migraine [1, 2]. Chronic migraine (CM), categorized using International Classification of Headache Disorders criteria [3], has a US prevalence of almost 1%, with the highest rates occurring in those aged in their 40s, in females, and in those with lower income [4]. CM is associated with high rates of headache-related disability and comorbidities [4, 5], placing a significant burden on affected individuals and ultimately leading to feelings of guilt, helplessness, or hopelessness about their migraine [6–8].

An intentional approach is essential to understanding the impact of therapeutic interventions in individuals with CM with comorbidities, including psychiatric conditions, like anxiety or depression which are highly prevalent among individuals with CM [9, 10]. People living with migraine who have comorbid anxiety and/or depression tend to face more severe migraine-related disability, a lower quality of life, and increased impairment in work productivity and daily activities [11]. It is crucial to understand the complex, bidirectional relationship, which may include underlying pathophysiology or genetic predisposition, between migraine and co-occurring psychiatric conditions and how they influence and exacerbate one another in an individual. Therefore, a more comprehensive understanding of treatment outcomes must consider this dynamic interplay. By acknowledging that these conditions influence each other, we can aim for a more robust holistic assessment of therapeutic effectiveness, leading to more tailored and effective care for individuals with migraine.

Brain fog has become more widely recognized in recent years as a symptom of several chronic conditions, but remains a somewhat nebulous and less studied symptom within the migraine population as data on brain fog are not commonly captured in clinical trials [12]. Brain fog can encompass a range of cognitive and higher-order thinking dysfunction, and the underlying neurochemical changes that occur during a migraine are known to impact perceivable cognitive function [13, 14]. Cognitive symptoms can occur both ictally and interictally, highlighting the presence of a significant symptom burden even on pain-free days [15–17]. Incorporating this understanding into meaningful clinical outcomes in therapeutic studies is important to better assess the holistic effectiveness of migraine treatments.

While clinical benchmarks such as number of monthly migraine days can provide a measure of migraine frequency and/or improvement [18, 19], individuals with migraine may also understand and appreciate terms and concepts such as “good days” or “crystal-clear days”, reflecting periods with minimal or no associated symptoms and greater daily functionality [15, 20]. This suggests a need for a more optimized and comprehensive approach to migraine care and assessment beyond the standard clinical benchmarks. Attention is turning toward a more holistic assessment of migraine that uses novel endpoints beyond reduction in migraine days, to try to reduce the overall burden and improve quality of life for individuals with migraine [21, 22].

The introduction of anti-calcitonin gene-related peptide (CGRP) monoclonal antibodies (mAbs) has helped to address a critical need in the prevention of migraine attacks and reduction of related symptoms and disability, with proven efficacy and tolerability in clinical trials [23–25]. As these therapies become more widely available, it is increasingly important to understand their impact in individuals with prior anti-CGRP mAbs exposure. Such insights are not available from clinical trials, which often exclude this population; therefore, real-world data on anti-CGRP mAbs like eptinezumab are vital for evaluating effectiveness in broader populations that more closely resemble those seen in clinical practice. Moreover, real-world evidence provides a way to assess the effectiveness and impact of switching within the drug class, such as from subcutaneous mAbs to intravenous (IV) eptinezumab [26, 27].

The REVIEW study was designed to report on the real-world experiences of individuals with CM being treated with eptinezumab in the outpatient setting. The results published to date showed that surveyed participants on average self-reported a two-fold increase in good days per month (from 8 to 18 good days/month) after starting treatment with eptinezumab, and a majority of participants expressed satisfaction with the effect of eptinezumab in reducing the severity, frequency, and duration of their migraine symptoms [20]. Furthermore, most participants reported satisfaction with eptinezumab impact on daily living, overall well-being, and improvements in brain fog [20]. Importantly, unlike most individuals with CM included in eptinezumab clinical trials to date, 89% of the participants in the REVIEW study had prior exposure to another anti-CGRP mAb [20]. The objective of this post hoc analysis of the REVIEW study was to further explore changes in good days per month in three subgroups of interest, those with psychiatric comorbidities, those with prior use of anti-CGRP mAbs, and those with the presence of brain fog.

## Methods

### Study design

REVIEW (Real-world EVidence and Insights into Experiences With eptinezumab) was an observational, multi-site study (4 tertiary headache centers) based in the United States that evaluated the real-world experiences of individuals being treated with eptinezumab for CM in the outpatient setting as well as the experiences of 4 treating principal investigators. The complete study methodology has been previously published [20]; in brief, the study consisted of a retrospective chart review, a structured participant survey, and a semi-structured healthcare provider interview. Data reported here are from the cross-sectional, post-eptinezumab participant survey.

Eligible participants were sorted by birth month (starting from January), and the first 25 participants from each site were selected to obtain consent for the survey part of the study. After obtaining consent, surveys were administered at the next infusion or office visit at three of the four sites, while one site administered participant surveys electronically.

### Participants

Study sites were instructed to select and recruit participants based on the key inclusion and exclusion criteria. Eligible participants (*n* = 94) were ≥ 18 years of age and had a diagnosis of CM per the participant chart. They were also required to have completed at least 2 full, consecutive eptinezumab infusion cycles (i.e., a minimum of 6 months’ exposure to eptinezumab), based on guidance from the American Headache Society (AHS) that advocate for a 6-month trial of quarterly therapies before assessing efficacy [28]. Individuals were excluded if they were treated with eptinezumab in a clinical trial or were currently enrolled in a migraine or headache clinical trial.

### Outcomes

The primary outcome of interest in this post hoc analysis was the participant-reported number of good days per month across subgroups evaluated. Participants specified the number (from 1 to 31) of good days per month both before and after initiating eptinezumab treatment. No definition of “good days” was provided, leaving interpretation to the participants’ discretion. Given the lack of a headache diary to capture the number of monthly migraine days, good days per month was considered a proxy for the participant-perceived effectiveness of eptinezumab.

Participants also reported improvement in brain fog, which was evaluated using 2 questions. The first asked: “Have you experienced ‘brain fog’ (feeling confused, have difficulty learning or remembering, or have trouble speaking or reading) before starting on eptinezumab?” Participants were requested to pick one response option: yes, no, or unsure. Participants who answered yes to the first question then proceeded to the second question: “If yes, please rate to what extent your symptoms have improved since starting eptinezumab.” Responses for this question were selected on a 5-point scale from “completely” to “not at all”.

### Subgroups analyzed

The participant-perceived effectiveness of eptinezumab (i.e., average number of good days per month) was analyzed in subgroups of interest: presence of a selected comorbid psychiatric condition; prior use of subcutaneously (SC) administered anti-CGRP mAb medications (number and type); and presence of brain fog. Additionally, good days per month were analyzed in subgroups defined by participant-rated extent of improvement in brain fog to evaluate the association between improvements in these outcomes.

The presence of a psychiatric condition was determined using a yes or no response. There were no inclusion/exclusion criteria for participants relating to psychiatric diagnoses or current symptomology, and no limitations on the number of specific diagnoses for each participant. Participants were able to select psychiatric comorbidities as an overarching category only and/or could select specific comorbidities of interest (anxiety, depression, bipolar disorder, and “other”). If “other” was selected, a free text option was provided, but adding text was not mandatory. Participants affirming a psychiatric diagnosis via the overarching category and/or a subcategory were included.

Subgroups based on the number or type of prior SC anti-CGRP mAb use were derived using a dual-choice (yes/no) question that asked if participants had ever received other preventive migraine therapy, and under “yes”, they were asked to specify which treatment(s) using a multiple-choice option. Among the preventive migraine medications listed as response options, the anti-CGRP mAbs included erenumab, fremanezumab, and galcanezumab. This information was necessary to distinguish between available preventive treatments, which have differing attributes and properties that may affect outcomes [29–32]: eptinezumab is administered IV, while the other 3 anti-CGRP mAbs are administered SC; erenumab is a fully human mAb and targets the CGRP receptor, while the other 3 anti-CGRP mAbs are humanized and target the CGRP ligand. Thus, subgroups based on the number of prior anti-CGRP mAbs included 0, 1, 2, or 3 prior treatments, and subgroups based on the type of prior anti-CGRP mAb used separated the individual treatments by ligand or receptor blockers (and were not mutually exclusive). Participants were not asked about the timing of anti-CGRP mAb use (e.g., initiation or discontinuation relative to eptinezumab); however, it was assumed that SC anti-CGRP mAb treatment was discontinued before switching to eptinezumab in accordance with typical clinical practice.

A self-reported history of brain fog before initiating eptinezumab treatment was determined using a yes or no response to the question “Have you experienced brain fog (feeling confused, have difficulty learning or remembering, or have trouble speaking or reading)” before starting eptinezumab treatment. Participants who responded yes were queried about the “extent your symptoms have improved” since starting eptinezumab treatment. Brain fog improvements were rated on a 5-point scale with the following response options: completely improved, very much improved, moderately improved, slightly improved, and not at all improved. The completely improved and very much improved ratings were grouped for analysis of the change from baseline (i.e., before eptinezumab treatment) in the participant-reported number of good days per month following at least 6 months’ exposure to eptinezumab.

### Statistical analysis

Data from all participants completing the one-time survey were included in this post hoc analysis. Observed data are reported for before and after eptinezumab treatment (i.e., no missing data imputation), and results were summarized descriptively. Summary statistics for continuous variables included the number of participants, mean, standard deviation, median, minimum, and maximum. For categorical variables, summary statistics included the absolute counts and percentages of participants with data (and counts of participants with missing data if applicable). Analyses were conducted using Microsoft Excel (version 10; Microsoft Corp., Redmond, WA), Stata BE (version 17; StataCorp, College Station, TX), R statistical software (version 4.3.1; R Core Team, Vienna, Austria), and SAS software (version 9.4 or higher; SAS Institute, Cary, NC).

## Results

### Study population

Full demographic information from the REVIEW study population has been previously published [20]. Overall, the study included 94 participants, of whom 78 (83%) were female, 84 (89%) were White or Caucasian, and 89 (95%) were not Hispanic nor Latino (Table S1). The mean age was ~ 49 years, and the mean time since CM diagnosis was ~ 15 years. All participants had previously received at least one prior preventive pharmacological therapy. Approximately half of the participants (*n* = 48, 51%) had received 5 or more eptinezumab infusions at the time of survey, with 54 participants (57%) reporting that 300 mg was the current dose level received [20].

**Fig. 1:**
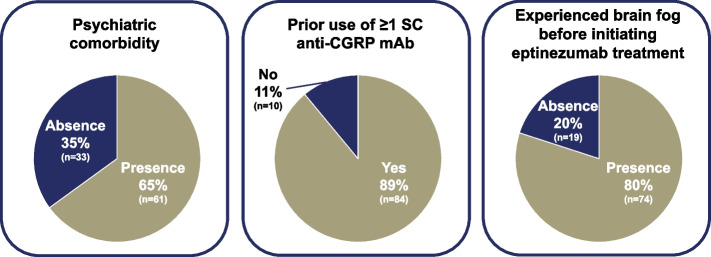
Percentage of each subgroup of interest out of the total REVIEW population (*N* = 94^a^). ^a^For brain fog before initiating eptinezumab treatment, only 93 participants answered the question. Anti-CGRP mAb, anti-calcitonin gene-related peptide monoclonal antibody; SC, subcutaneous

There were 61 participants (65%) who reported the presence of psychiatric comorbidities, including anxiety (*n* = 47; 50%), depression (*n* = 45; 48%), bipolar disorder (*n* = 3; 3%), and “other” (*n* = 4; 4%), with participants able to select more than one response. There were 84 participants (89%) who reported prior use of ≥ 1 SC anti-CGRP mAb, with erenumab the most common (*n* = 65), followed by galcanezumab (*n* = 52), and fremanezumab (*n* = 49). Subgroups who had received 0, 1, 2, or 3 prior SC anti-CGRPs mAbs included 10 (11%), 27 (29%), 32 (34%), and 25 (27%) participants, respectively. Among the 93 participants who answered the initial survey question about brain fog, 74 (80%) reported experiencing brain fog before initiating eptinezumab treatment (Fig. 1).

### Good days per month in subgroups of interest

In the total REVIEW participant population, mean good days per month increased from 8 before initiating eptinezumab to 18 after initiating eptinezumab, representing a more than twofold increase [20].

**Fig. 2:**
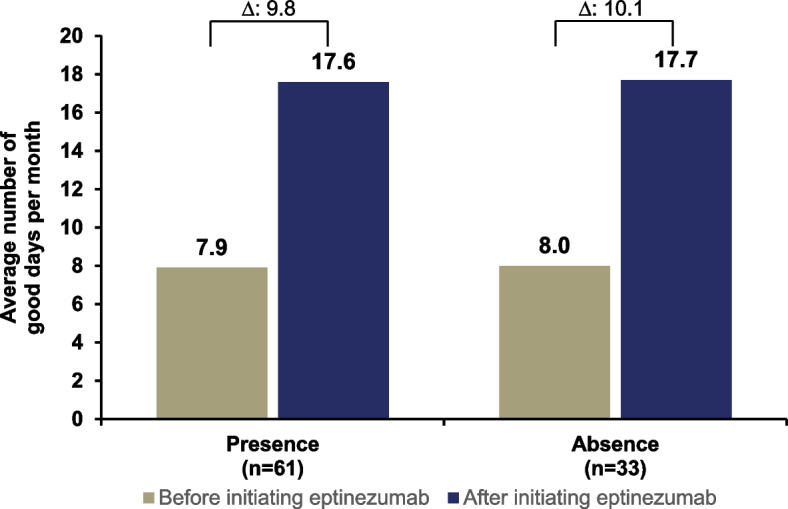
Self-reported good days/month before and after eptinezumab by presence/absence of psychiatric condition(s). *N* = 94. Individual group means and the calculated mean difference are rounded separately; the mean change from baseline was first estimated at the individual level, and the mean delta was then calculated, which may result in minor discrepancies. Participants reported the presence (answer: yes) or absence (answer: no) of psychiatric conditions at baseline. Participant prompt: “On average, how many good days per month did you experience before/after starting on eptinezumab? Please indicate the number of days, 1–31.”

Participant-reported good days after initiating eptinezumab treatment were similar regardless of the presence (17.6 days; participants who answered yes, *n* = 61) or absence (17.7 days; participants who answered no, *n* = 33) of psychiatric conditions at baseline (Fig. 2). The average change from baseline in good days per month was also similar between the two groups (presence at baseline: 9.8 days [95% CI, 7.4 to 12.1] and absence at baseline: 10.1 days [95% CI, 6.6 to 13.7]) (Fig. 2).

**Fig. 3:**
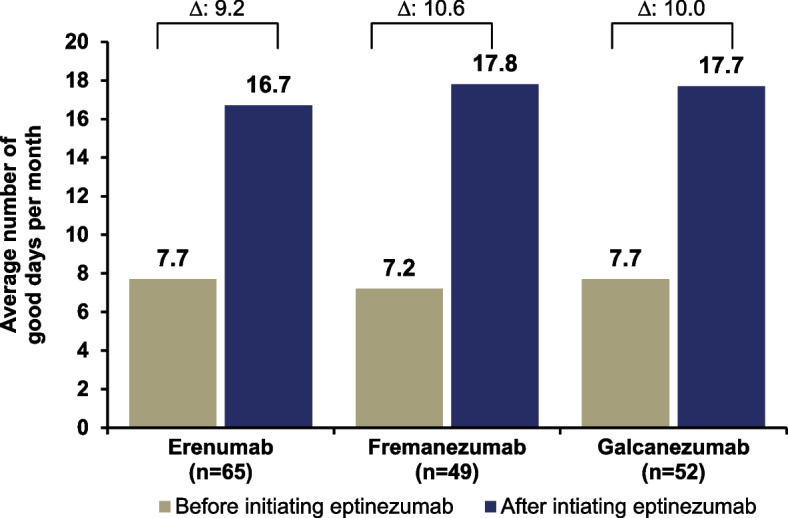
Self-reported good days/month before and after eptinezumab by prior type of subcutaneous anti-CGRP mAb. Individual group means and the calculated mean difference are rounded separately; the mean change from baseline was first estimated at the individual level, and the mean delta was then calculated, which may result in minor discrepancies. Participant prompt: “On average, how many good days per month did you experience before/after starting on eptinezumab? Please indicate the number of days, 1–31.” Subgroups were not mutually exclusive; participants could be included in more than one group. In addition, we did not elicit information on whether the good days per month prior to starting eptinezumab were whilst using a prior mAb or during a washout period. Anti-CGRP, anti-calcitonin gene-related peptide; mAb, monoclonal antibody

Among the 84 participants who reported that they had previously received ≥ 1 SC anti-CGRP mAb before starting eptinezumab treatment, the number of good days per month more than doubled following eptinezumab initiation regardless of whether the prior exposure was to a CGRP receptor blocker (16.7 days, erenumab) and/or a ligand blocker (17.8 days, fremanezumab; 17.7 days, galcanezumab) (Fig. 3). The average change from baseline in good days per month was also comparable between participants regardless of the type of prior SC anti-CGRP mAb used (erenumab, 9.2 days [95% CI, 6.7 to 11.6]; fremanezumab, 10.6 days [95% CI, 8.1 to 13.1]; galcanezumab, 10.0 days [95% CI, 7.0 to 12.9]) (Fig. 3).

**Fig. 4:**
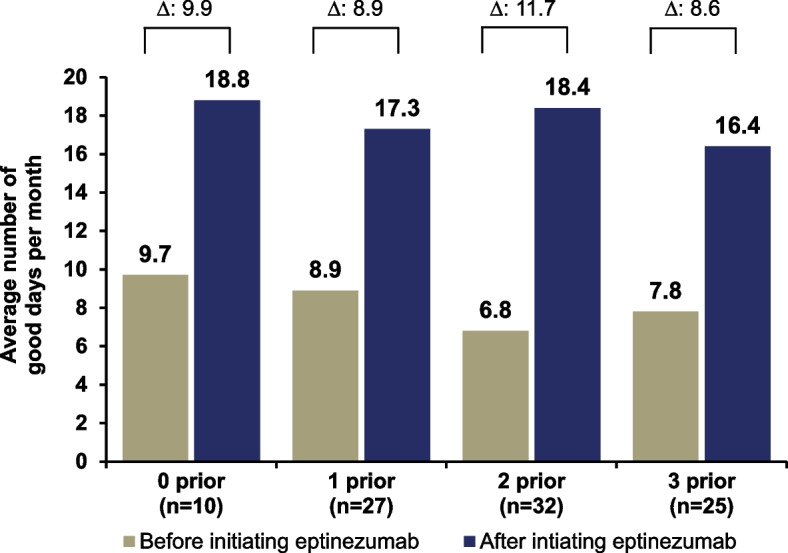
Self-reported good days/month before and after eptinezumab by number of prior subcutaneous anti-CGRP mAbs. Individual group means and the calculated mean difference are rounded separately; the mean change from baseline was first estimated at the individual level, and the mean delta was then calculated, which may result in minor discrepancies. Participant prompt: “On average, how many good days per month did you experience before/after starting on eptinezumab? Please indicate the number of days, 1–31.” Anti-CGRP, anti-calcitonin gene-related peptide; mAb, monoclonal antibody

Regardless of the number of prior SC anti-CGRP mAbs used, the number of good days per month approximately doubled following eptinezumab initiation (0 prior, 18.8 days; 1 prior, 17.3 days; 2 prior, 18.4 days; 3 prior, 16.4 days) (Fig. 4) with the average change from baseline in good days per month by number of prior SC anti-CGRP mAb treatments being similar between participants (0 prior, 9.9 days [95% CI, 5.4 to 14.3]; 1 prior, 8.9 days [95% CI, 5.5 to 12.3]; 2 prior, 11.7 days [95% CI, 8.0 to 15.4]; 3 prior, 8.6 days [95% CI, 4.6 to 12.6]) (Fig. 4).

**Fig. 5:**
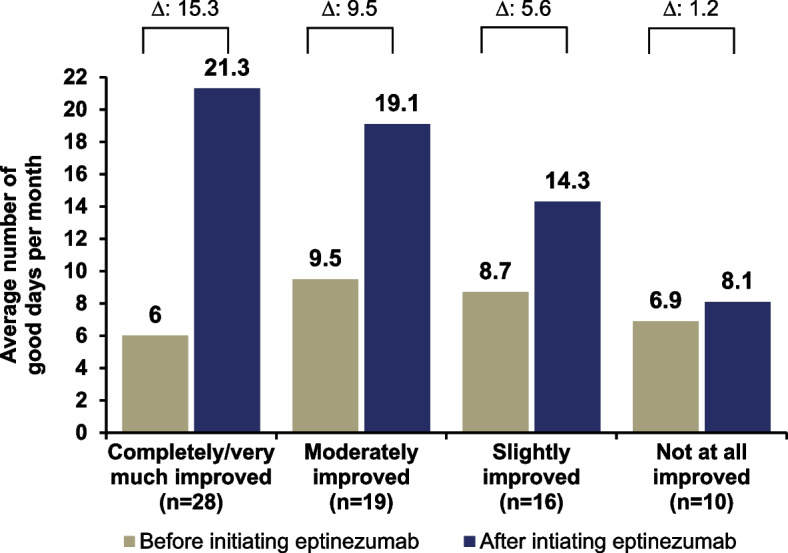
Self-reported good days/month before and after eptinezumab stratified by extent of brain fog improvement. Individual group means and the calculated mean difference are rounded separately; the mean change from baseline was first estimated at the individual level, and the mean delta was then calculated, which may result in minor discrepancies. Participant prompts: “Have you experienced ‘brain fog’ (feeling confused, have difficulty learning or remembering, or have trouble speaking or reading) before starting on eptinezumab?” “If yes, please rate to what extent your symptoms have improved since starting eptinezumab. Please place a checkmark in the box which most closely fits your opinion.”

Overall, eptinezumab treatment showed similar increases in number of good days per month between participants with (presence) and without (absence) brain fog (18.8 days and 17.4 days, respectively) (Fig. S1). Among the 74 participants who reported experiencing brain fog before initiating eptinezumab treatment, 28 (38%) rated their symptoms as completely/very much improved, 19 (26%) as moderately improved, 16 (22%) as slightly improved, and 10 (14%) as not at all improved; 1 participant did not provide a response. Those who reported significant improvement (completely/very much improved) in their brain fog symptoms after eptinezumab initiation also reported a 15-day increase in number of good days per month, while participants who reported no improvement in brain fog reported a 1-day increase (Fig. 5). The average change from baseline in good days per month was larger in subgroups with greater improvements in brain fog: completely/very much improved, 15.3 [95% CI, 11.9 to 18.6]; moderately improved, 9.5 [95% CI, 7.3 to 11.7]; slightly improved, 5.6 [95% CI, 2.6 to 8.6]; not at all improved, 1.2 [95% CI, −1.4 to 3.8] (Fig. 5).

## Discussion

The findings from this post hoc analysis of the REVIEW study data show that the effectiveness of eptinezumab, as measured by an increase in good days per month in real-world settings, remained consistent and unaffected across three key subgroups of interest: participants with psychiatric comorbidities, those with prior exposure to anti-CGRP mAbs, and those experiencing brain fog. These results underscore the effectiveness of eptinezumab across a variety of commonly encountered subgroups of individuals with CM in real-world clinical settings and can provide clinicians with greater confidence when selecting eptinezumab for the populations they manage—particularly those with similar characteristics and backgrounds to our study subgroups.

Individuals with migraine too often face stigma [33, 34], and this can be further exacerbated in those with comorbid psychiatric conditions [35]. Such negative perceptions of migraine increase the burden on individuals with migraine and may hinder their access to treatment and support [36]. Historically, there has been a misperception that individuals with both migraine and psychiatric symptoms were likely to be “difficult to treat,” with a lower likelihood of responding to therapy [35], and this misperception may be more pronounced in individuals with higher burden of disease such as CM. However, this view has not been substantiated by data, in fact studies show that various forms of preventive therapy including behavioral therapy, traditional oral preventive therapies, onabotulinumtoxinA, and CGRP-targeting mAbs can be effective in this population [37, 38]. Our data adds to this body of evidence, indicating that the presence of psychiatric conditions such as depression or anxiety did not impact the effectiveness of eptinezumab in individuals with CM in a real-world setting. Participants in REVIEW, with and without psychiatric conditions, responded equally well to eptinezumab treatment, according to the self-reported good days per month outcome. These findings offer meaningful guidance to clinicians managing people living with CM and suggest that the presence of psychiatric comorbidities should not deter the selection of anti-CGRP monoclonal antibodies. While these agents do not directly treat psychiatric conditions, the observed clinical benefits in individuals with comorbid psychiatric disorders underscore their value in this population.

Another key outcome from our analysis was that in this population of adults with CM, neither the type of prior SC anti-CGRP mAb (receptor blocker-erenumab; ligand blockers-galcanezumab, or fremanezumab) nor the number of prior SC anti-CGRP mAbs used (0, 1, 2, or 3) impacted the effectiveness of eptinezumab. While anti-CGRP mAbs are becoming more widely used in CM treatment, there remains the important clinical question of whether the mechanism of action (ligand or receptor blocker) may correlate with differences in effectiveness [39], and how prior treatment with a SC-administered anti-CGRP mAb might impact the effectiveness of IV-administered eptinezumab [40, 41]. The response we observed in the REVIEW study, whereby the average number of good days per month doubled after eptinezumab initiation, was irrespective of the mechanism of CGRP blockade of previously used SC anti-CGRP mAb(s), indicating that exposure to a previous ligand or receptor-targeted therapy did not impact the effectiveness of eptinezumab. In addition, the effectiveness of eptinezumab in increasing the number of good days per month did not differ based on the number of previous SC anti-CGRP mAbs used, suggesting that a positive response can be attained at various timepoints within the treatment journey.

Individuals with CM frequently undergo a long and often thwarted journey before being prescribed effective treatment including medication [42]. Earlier use of eptinezumab within the treatment plan could both minimize excessive exposure to multiple treatments (both anti-CGRP mAbs and other drug classes) and avoid the common practice of cycling through treatments in search of efficacy. This approach moves away from the commonly adopted practice of sequentially trialing a set of therapies based on anecdotal assumptions before considering more effective treatments. Thus, our data support practice considerations that discourage an unnecessarily prolonged, stepwise approach of multiple treatment failures before transitioning to eptinezumab, and align with the recent position statement/guideline from societies including the AHS, European Headache Federation, and Canadian Headache Society, which advocate the first-line use of CGRP-targeting therapies for migraine prevention [43–45].

Currently, clinical trials in migraine prevention use endpoints such as responder rates and reductions in monthly migraine days, based on eDiary data [46]; however, recent machine learning data from the population-based OVERCOME survey found that moderate/severe migraine-related disability, moderate/severe migraine interictal burden, and severe allodynia were most associated with seeking care for headache/migraine [47]. Therefore, in the real world, perceptions of treatment effectiveness must account for the holistic burden of migraine, which can require more subjective and patient-centric measures, such as good days per month [15, 20, 48, 49]. In addition, this highlights a need for regulatory bodies to explore opportunities to better elucidate the holistic burden of migraine when evaluating treatment efficacy in clinical trials. The REVIEW study aimed to assess effectiveness according to the number of good days per month as reported by participants; although the interpretation of what constitutes a good day was left to the interpretation of each participant, which can allow for inclusion of both objective factors (e.g., presence/absence of migraine pain) and subjective factors (e.g., perception of functionality and life fulfillment as it relates to having CM) important to the individual.

There is a growing understanding that brain fog and other cognitive impairment is a prevalent and burdensome part of the migraine experience [8, 16, 50], although definitions for these symptoms are varied. At present, data and research into the impact of anti-CGRPs mAbs on brain fog are limited [51]. Notably, participants in REVIEW who reported complete/very much improvement in brain fog had an average 15-day increase in the number of good days per month, while a 1-day increase was reported in those whose brain fog did not improve, demonstrating a correlation between the degree of brain fog improvement and the number of participant-reported good days per month. Furthermore, the association between brain fog and increase in number of good days per month demonstrates that cognitive impairment may be an important element in the way individuals report migraine improvements (i.e., what constitutes as a good day). Our findings suggest that good days per month may serve as a meaningful real-world proxy for the holistic burden of migraine and highlight the need for further research in this area.

The main limitations of this report included the participant-reported nature of the data collection (which is dependent on a retrospective reporting of information that may have resulted in a recall bias for all evaluated outcomes), the relatively small survey population (i.e., 94 individuals in headache specialist/neurology settings), the inherent selection bias due to the requirement for participants to have had two doses of eptinezumab (i.e., 6 months of exposure), and the nature of the analyses (i.e., post hoc). Many questions were multiple choice, were administered with no additional guidance or information provided, and were subject to individual interpretation. In particular, the term “good days” was not defined and is subjective and likely interpreted in different ways by different individuals; therefore, only participants who reported a response before and after eptinezumab treatment were included in the analysis. In addition, information relating to change in brain fog was only collected in terms of improvement and response options were not provided to assess the worsening of brain fog; therefore, we were able to evaluate brain fog only in terms of degrees of improvement, rather than worsening or overall change. As the survey data were self-reported, and dependent on participant recall, it is possible that faulty or incomplete information could have been provided by participants who had undergone several eptinezumab infusions over months or years and could no longer accurately remember details of their pre-eptinezumab status. Participants were not asked about the timing of anti-CGRP mAb use (i.e., initiation or discontinuation relative to eptinezumab); however, it was assumed that SC mAb treatment was discontinued before switching to eptinezumab in accordance with typical clinical practice. In addition, the reasons for switching from SC treatments to eptinezumab cannot be determined from this study. While the brain fog questions were framed within the context of migraine, the questions were not specific into assessing frequency, severity, or timing (i.e., ictal, interictal, or both; always or occasional; recently or at any time in the past) during the pre-eptinezumab period. Furthermore, it would be difficult for patients to delineate whether brain fog was attributed to migraine or several other conditions and/or medications. There was no question asking whether participants thought their brain fog was most likely to be related to their migraine, migraine treatment, or another condition (such as coronavirus disease 2019 infection, pregnancy, menopause, or depression), and we did not ask when the brain fog improved in relation to eptinezumab initiation, or whether improvements were intermittent or continuous.

## Conclusions

This post hoc analysis of REVIEW provides much-needed information on the lived experience of individuals with CM who are being treated in clinical practice today. Our data support the strong effectiveness of eptinezumab across all subgroups evaluated, regardless of presence of psychiatric comorbidities, prior anti-CGRP mAb treatments, or in those who experience brain fog. Our findings challenge the concept of the "difficult to treat patient" and demonstrate that these patients also respond successfully to preventive management. Clinicians treating individuals with migraine can have confidence that eptinezumab can be effective when initiated in individuals with psychiatric comorbidities, in those who have previously tried other anti-CGRP mAbs, and/or in those who experience brain fog. Additionally, as our understanding of migraine evolves, we must continue to explore novel ways for characterizing the multidimensional burden of the disorder and for evaluating therapeutic effectiveness across diverse clinical domains.

## Supplementary Information

Supplementary Material 1: Table S1. Selected baseline characteristics of REVIEW participants.^a^Subgroups were not mutually exclusive; participants could be included in more than one group. ^b^One participant self-reported only receiving 1 eptinezumab infusion: however, all participants included in the study had ≥ 2 infusions, confirmed by the physician and chart review. Anti-CGRP, anti-calcitonin gene-related peptide; mAb, monoclonal antibody. Fig. S1. Self-reported good days/month before and after eptinezumab stratified by presence/absence of brain fog. Individual group means and the calculated mean difference are rounded separately; the mean change from baseline was first estimated at the individual level, and the mean delta was then calculated, which may result in minor discrepancies. While 74 and 19 participants, respectively, reported the presenceand absenceof brain fog, only 73 and 18 had evaluable data for good days per month. Participant prompts: “Have you experienced ‘brain fog’before starting on eptinezumab?” Answer options “Yes” or “No.”

## Data Availability

The data sets generated and/or analyzed during the current study are not publicly available due to data license restrictions. However, summarized data are available from the corresponding author upon reasonable request.

## Abbreviations

AHS: American Headache Society
CGRP: Calcitonin gene-related peptide
CM: Chronic migraine
IV: Intravenous
mAb: Monoclonal antibody
REVIEW: Real-world EVidence and Insights into Experiences With eptinezumab
SC: Subcutaneous
