# Supplementary material for: Real-world effectiveness of eptinezumab in chronic migraine—increase in good days in three subgroups: psychiatric comorbidities, prior subcutaneous anti-calcitonin gene-related peptide therapy, and migraine-associated brain fog
Source: J Headache Pain. 2025 Nov 27;26(1):274. doi: 10.1186/s10194-025-02165-2 (PMC12659521; doi:10.1186/s10194-025-02165-2)
Supplement: Supplementary file 1 — Supplementary Material 1: Table S1. Selected baseline characteristics of REVIEW participants.aSubgroups were not mutually exclusive; participants could be included in more than one group. bOne participant self-reported only receiving 1 eptinezumab infusion: however, all participants included in the study had ≥ 2 infusions, confirmed by the physician and chart review. Anti-CGRP, anti-calcitonin gene-related peptide; mAb, monoclonal antibody. Fig. S1. Self-reported good days/month before and after eptinezumab stratified by presence/absence of brain fog. Individual group means and the calculated mean difference are rounded separately; the mean change from baseline was first estimated at the individual level, and the mean delta was then calculated, which may result in minor discrepancies. While 74 and 19 participants, respectively, reported the presenceand absenceof brain fog, only 73 and 18 had evaluable data for good days per month. Participant prompts: “Have you experienced ‘brain fog’before starting on eptinezumab?” Answer options “Yes” or “No.” [file 10194_2025_2165_MOESM1_ESM.pdf]

SUPPLEMENTAL MATERIAL

Table S1 Selected baseline characteristics of REVIEW participants

Fig. S1 Self-reported good days/month before and after eptinezumab stratified by presence/absence of brain fog

Table S1 Selected baseline characteristics of REVIEW participants (self-reported)

	**Participants (N=94)**
**Gender: Female, n (%)**	78 (83)
**Age (years), mean**	49.2
**Race, n (%)**	
White or Caucasian	84 (89)
Black or African American	8 (9)
Asian	1 (1)
Other	1 (1)
**Ethnicity: Non-Hispanic or -Latino, n (%)**	89 (95)
**Time since migraine diagnosis (years), mean**	15.4
**Diagnosis of psychiatric comorbidities, n (%)[a]**	
Anxiety	47 (50)
Depression	45 (48)
Bipolar disorder	3 (3)
Other	4 (4)
**Previous type of anti-CGRP mAb preventive therapy, n (%)[a]**	
Erenumab	65 (69)
Fremanezumab	49 (52)
Galcanezumab	52 (55)
**Number of prior subcutaneous anti-CGRP mAb, n (%)**	
0	10 (11)
1	27 (29)
2	32 (34)
3	25 (27)
**Number of eptinezumab infusions received, n (%)**	
1[b]	1 (1)
2	12 (13)
3	15 (16)
4	17 (18)
5 or more	48 (51)
Missing	1 (1)
**Current eptinezumab dose received, n (%)**	
100 mg	22 (23)
300 mg	54 (57)
Other	2 (2)
Cannot recall	13 (14)
Missing	3 (3)

[a]Subgroups were not mutually exclusive; participants could be included in more than one group.

[b]One participant self-reported only receiving 1 eptinezumab infusion: however, all participants included in the study had ≥ 2 infusions, confirmed by the physician and chart review.

Anti-CGRP, anti-calcitonin gene-related peptide; mAb, monoclonal antibody.

Fig. S1 Self-reported good days/month before and after eptinezumab stratified by presence/absence of brain fog

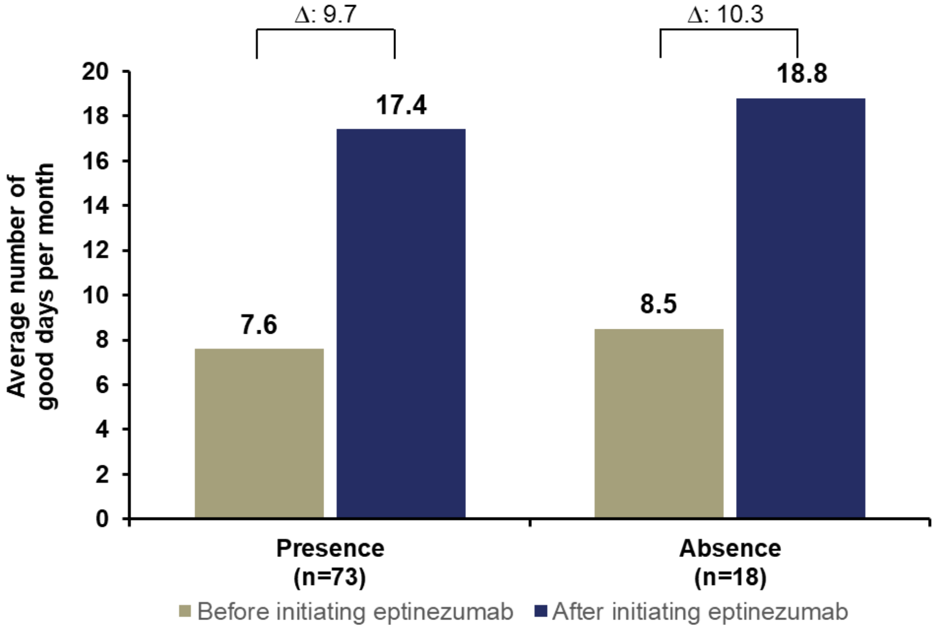

Individual group means and the calculated mean difference are rounded separately; the mean change from baseline was first estimated at the individual level, and the mean delta was then calculated, which may result in minor discrepancies. While 74 and 19 participants, respectively, reported the presence (yes) and absence (no) of brain fog, only 73 and 18 had evaluable data for good days per month.

Participant prompts: “Have you experienced ‘brain fog’ (feeling confused, have difficulty learning or remembering, or have trouble speaking or reading) before starting on eptinezumab?” Answer options “Yes” or “No.”
